# Supplementary material for: Gay Community Stress Scale with Its Cultural Translation and Adaptions in Taiwan
Source: Int J Environ Res Public Health. 2022 Sep 15;19(18):11649. doi: 10.3390/ijerph191811649 (PMC9517012; doi:10.3390/ijerph191811649)
Supplement: Supplementary file 1 [file ijerph-19-11649-s001.zip › ijerph-1872362-supplementary.pdf]

Supplementary Table

Table S1. The traditional Chinese version of the Gay Community Stress Scale.

繁體中文版男同志族群壓力量表

以下是有些人認為<u>主流男同志族群</u>有這樣的的觀點，當然不一定每個人都這麼覺得，請就你實際生活中對男同志族群的了解，回答它對你在現實生活中造成的壓力或困擾有多高。

正式量表題號	原始量表題號		這句話描述的主流男同志社群觀點，在現實生活中對你造成<u>壓力或困擾</u>的程度為何?				
			完全沒有困擾	有一點困擾	中等程度困擾	相當嚴重困擾	非常嚴重困擾
1	1	主流男同志族群重視性多過於深入的人際關係。	1	2	3	4	5
2	2	在主流男同志族群中，要維持感情關係是有困難的。	1	2	3	4	5
3	3	主流男同志族群過度關注性(sex)這件事。	1	2	3	4	5
4	4	在主流男同志族群之中，人人彼此都有發生過性行為。	1	2	3	4	5
5	5	主流男同志族群太過於沈迷在約炮和約會軟體。	1	2	3	4	5
6	6	主流男同志族群中很常發生危險性行為(像無套肛交)。	1	2	3	4	5
7	7	主流男同志族群過度看重一個人要有很好的工作。	1	2	3	4	5
8	8	在主流男同志族群中，富有的人比較被重視。	1	2	3	4	5
9	9	主流男同志族群極度看重有權有勢的人。	1	2	3	4	5
10	10	主流男同志族群極度看重時髦穿著以及最新時尚。	1	2	3	4	5
11	11	在主流男同志族群中有許多爭執、口角、陰險詭計。	1	2	3	4	5
12	12	主流男同志族群非常熱衷八卦、說他人是非。	1	2	3	4	5
13	13	主流男同志族群盛行著競爭和忌妒的文化。	1	2	3	4	5
14	14	主流男同志族群非常愛搞小圈圈。	1	2	3	4	5
15	15	在主流男同志族群中，朋友之間有許多猜忌、不信任。	1	2	3	4	5
16	16	主流男同志族群極度愛評斷他人。	1	2	3	4	5
17	17	主流男同志族群過度強調金錢和物質。	1	2	3	4	5
18	18	主流男同志族群常有著種族或族群偏見。	1	2	3	4	5
19	19	主流男同志族群總是把有色人種跟性聯想在一起。	1	2	3	4	5
20	20	主流男同志族群會排斥感染愛滋病毒的男同志。	1	2	3	4	5

21	21	主流男同志族群過度看重健美的身材。	1	2	3	4	5
22	22	主流男同志族群非常看重陰莖的尺寸。	1	2	3	4	5
23	23	主流男同志族群過度看重陽剛氣質。	1	2	3	4	5
24	24	主流男同志族群認為老男人比較沒人要。	1	2	3	4	5
25	26	主流男同志族群過度瘋迷社交媒體，例如臉書、推特、IG。	1	2	3	4	5
26	28	主流男同志族群太愛把人歸到某個特殊類型，例如花美男、熊、運動型等。	1	2	3	4	5
27	29	主流男同志族群極度關注性愛姿勢，像是 1、0、不分。	1	2	3	4	5
28	30	主流男同志族群認為沒向家人出櫃的人是魯蛇。	1	2	3	4	5
29	32	主流男同志族群看輕有身體疾病的人。	1	2	3	4	5
30	33	主流男同志族群看輕有精神疾病的人。	1	2	3	4	5
31	34	主流男同志族群看輕原住民。	1	2	3	4	5
32	35	主流男同志族群看輕住在鄉鎮、不熱鬧地區的人。	1	2	3	4	5

Construct, items and scoring

Mean score of the Sex construct: dividing the total scores of items 1 to 6 by 6

Mean score of the Status construct: dividing the total scores of items 7, 8, 9, 10 and 17 by 5

Mean score of the Competition construct: dividing the total scores of items 11 to 16 by 6

Mean score of the Exclusion construct: dividing the total scores of items 18, 19, 20, 28, 29, 30, 31 and 32 by 8

Mean score of the Externals construct: dividing the total scores of items 21, 22, 23, 24, 25, 26 and 27 by 7
